# Use of Innovative Methods to Produce Highly Insulating Walls Using 3D-Printing Technology

**DOI:** 10.3390/ma17163990

**Published:** 2024-08-11

**Authors:** Michał Góra, Magdalena Bańkosz, Bożena Tyliszczak

**Affiliations:** 1 Department of Materials Engineering, Faculty of Materials Engineering and Physics, Cracow University of Technology, 37 Jana Pawla II Av., 31-864 Krakow, Poland; michal.gora@doktorant.pk.edu.pl; 2 4ROBOT Sp. z o.o., 15 Tadeusza Kościuszki, 32-650 Kęty, Poland

**Keywords:** 3D-printing technology, insulating materials, optimizing thermal properties

## Abstract

The article explores innovative methods for creating high-insulation walls, essential for the future of energy-efficient and sustainable construction. It focuses on advanced 3D-printing technologies that allow for the construction of walls with superior insulation materials, optimizing thermal properties and significantly reducing energy for heating and cooling. The integration of thermal insulation within wall structures and innovations in building materials like lightweight composites, aerogels, and nanotechnology-based insulations are highlighted. It discusses the environmental, economic, and technical benefits of these innovations and the challenges to fully leverage 3D printing in construction. Future development directions emphasize materials that enhance thermal efficiency, sustainability, and functionality, promising a new era of sustainable and innovative construction practices.

## 1. Introduction

Thermal insulation in construction is a crucial aspect aimed at ensuring optimal thermal conditions inside buildings translating into user comfort and energy efficiency of the entire structure. This work focuses on minimizing unwanted heat flows between the interior of a building and its surroundings, which is essential for maintaining a stable and desired temperature inside rooms regardless of external conditions. Introducing this issue involves understanding the basic principles of thermodynamics, including heat transfer methods: conduction, convection, and radiation [1]. The key here is the use of materials with a suitably low thermal conductivity coefficient, capable of effectively insulating the interior of the building, reducing heat loss in winter and excessive temperature increase in summer. The issue of thermal insulation also includes the analysis of thermal bridges, i.e., places in the building structure through which heat escapes faster than through other structural elements, leading to local heat losses and potentially causing problems with humidity or mold. Solving these problems requires careful design and execution, as well as the use of special insulation techniques [2,3]. In the context of modern construction, thermal insulation is also linked to the goal of building passive or energy-efficient buildings that use advanced insulation systems along with intelligent design to minimize the energy demand for heating or cooling. This, in turn, is associated with a wide range of insulation materials, from traditional ones like mineral wool or styrofoam to modern solutions such as vacuum panels or polyurethane foams [4]. The introduction to the issue of thermal insulation in construction highlights its complexity and multidimensionality, pointing to the need for an interdisciplinary approach that combines knowledge from the fields of building physics, architecture, material engineering, and ecology. It is an area of continuous research and innovation, aimed not only at improving the quality of life but also at reducing the impact of construction on the natural environment [5]. The importance of innovative manufacturing methods in the context of increasing the energy efficiency of buildings is crucial, as these modern technologies and approaches enable a significant reduction in energy consumption and emissions of harmful substances into the atmosphere. By using advanced building materials, such as high-performance thermal insulations, intelligent building management systems (BMSs), or renewable energy technologies, it is possible to significantly increase the energy efficiency of buildings [6,7]. Innovative manufacturing methods, such as 3D printing in construction, allow for creating more complex geometries of buildings and structural elements, which can contribute to better management of air and light flows, thereby increasing natural heating and lighting. Additionally, the use of advanced technologies can shorten construction time and reduce waste, thus lowering the overall carbon footprint of the building [8]. Innovative approaches, such as modular prefabricated construction, allow for more precise quality control of manufactured elements, leading to greater airtightness of buildings and smaller energy losses. Additionally, the development and implementation of smart solutions, such as intelligent windows reacting to changing external conditions or AI-based energy management systems, allow for real-time optimization of energy consumption, adapting to user needs and weather conditions [9,10].

## 2. Overview of 3D-Printing Technology

### 2.1. History and Development of 3D Printing in Construction

**Figure 1:**
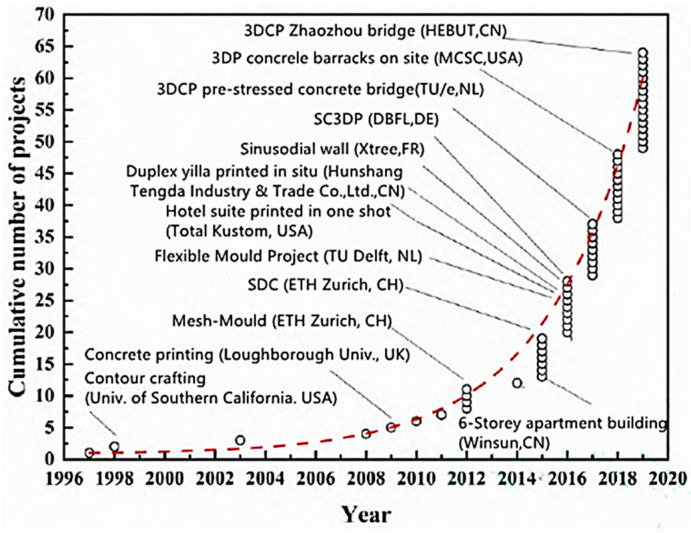
Development history of 3D-printing construction technology [14].

The history and evolution of 3D printing in the construction sector began with the first experiments in additive manufacturing in the 1980s, evolving to today’s innovative applications such as buildings made of 3D-printed concrete or prefabricated construction components [6,10,11]. Three-dimensional printing, also known as additive manufacturing (AM), allows for the precise creation of complex geometries that are difficult or even impossible to achieve using traditional construction methods. Technologies such as concrete printing, Fused Deposition Modeling (FDM) for plastics, and even metal printing open up new possibilities in designing highly thermally insulated walls using a variety of materials. In the context of producing highly insulated walls, the key factor is the choice of insulation materials that can be effectively used in 3D printing [12,13]. The development of 3D printing methodologies used in construction is presented in Figure 1.

Materials such as geopolymers, lightweight insulating concretes, and innovative composites containing aerogels may offer exceptional insulating properties. Three-dimensional printing is a tool that not only enables innovations in design and construction but also contributes to sustainable development in the construction industry [15,16]. Three-dimensional printing in construction is based on the fundamental principles of additive manufacturing, where an object is built by successively laying down layers of material. This method allows for the production of complex shapes and structures that are difficult or impossible to achieve with traditional construction techniques. Among the 3D-printing technologies used in construction, the most popular are FDM, SLS (Selective Laser Sintering), and SLA (Stereolithography), each offering unique possibilities and material properties [14]. Three-dimensional printing offers many benefits, such as reducing construction waste, increasing construction precision, and the potential for design innovation. However, challenges such as high initial costs, the need for specialized knowledge, and scale limitations may hinder the widespread adoption of this technology [17]. The future of 3D printing in construction may include the development of new materials with better insulating properties, integration with intelligent building systems, and further automation of the construction process. It is also important to take steps towards standardization and regulation to facilitate broader adoption of this technology [18,19].

### 2.2. Basic Principles and Types of 3D-Printing Technologies Used in Construction

Three-dimensional-printing technology in construction is based on the fundamental principles of additive manufacturing, where an object is created by successively laying down layers of material. This method allows for the production of complex shapes and structures that are difficult or impossible to achieve using traditional construction methods. Among the 3D-printing technologies used in construction, the most popular are FDM (Fused Deposition Modeling), SLS (Selective Laser Sintering), and SLA (Stereolithography), each offering unique capabilities and material properties. A comparison between traditional methods and 3D printing in construction is presented in Table 1.

### 2.3. Fused Deposition Modeling (FDM)

**Figure 2:**
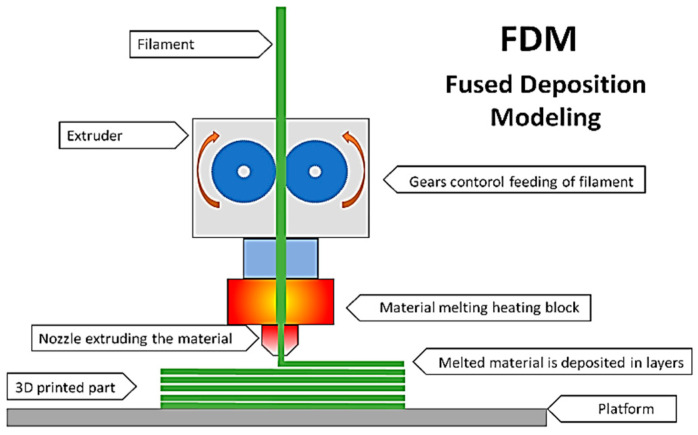
A diagram of the 3D-printing process using the FDM technique [23].

Fused Deposition Modeling (FDM) is a 3D-printing technique that significantly contributes to the popularization of additive manufacturing. This technique allows for the rapid and cost-effective creation of prototypes and functional parts. In most cases, when using the FDM technique, the costs of consumable materials are significantly lower compared to the materials used in other 3D-printing methods, which reduces the overall production cost. Furthermore, FDM equipment is relatively inexpensive and widely available, allowing for quick amortization of the investment [13]. Additionally, the FDM technology enables rapid prototyping due to its relatively simple and fast process of material layer deposition, which accelerates design iterations and shortens time to market. Moreover, FDM is an additive method that minimizes material waste compared to traditional subtractive methods, which also contributes to cost reduction [20,21]. In construction, FDM is primarily used for creating architectural models, installation components, and decorative elements [22]. The use of the FDM technique is shown in Figure 2.

**Figure 3:**
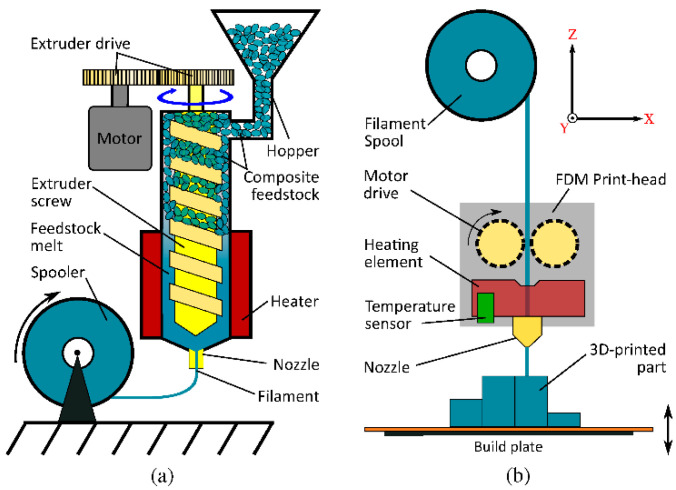
(**a**) A schematic depiction of the filament extrusion process. The kneaded composite material chunks are forced down a heated barrel using a screw, extruded through a nozzle of a defined diameter and spooled. (**b**) The FDM printing process: A motor drive forces the filament into the temperature-controlled print-head, mounted on an XY stage. After being forced through a nozzle, the material is deposited on to a build plate, which moves down to allow for the structuring of the next layer [25].

Due to its accessibility and ease of use, FDM is a popular tool among architects and engineers for visualizing projects and testing concepts. It involves extruding melted thermoplastic material through a moving nozzle, which precisely deposits the material layer by layer, building the object from the bottom up. The process starts with a digital 3D model, which is sliced into thin sections by specialized software. Then, each layer is reproduced on the printer’s platform by melting and extruding the material from the extruder nozzle, precisely controlled based on the 3D model data. FDM utilizes a variety of thermoplastics, such as ABS, PLA, PETG, each offering unique properties, allowing for the material to be tailored to specific design requirements [24]. This technique is characterized by affordable costs and simplicity of use, making it popular among DIY enthusiasts, in education, as well as in professional prototyping and production applications. However, despite its many advantages, FDM also has its limitations. The process of filament extrusion occurring in the FDM technique is presented in Figure 3.

Using FDM, walls can be precisely created layer by layer, allowing the implementation of complex structures with integrated channels or air spaces that improve insulation properties. The process allows the integration of materials with different properties, such as filaments with nanotechnology additives or aerogels, which increase insulation efficiency by eliminating the need for additional application of insulation materials [26,27]. With the additive approach characteristic of FDM, material waste is minimized, resulting in more sustainable production and reduced costs. The technology also allows for customizable printing parameters, such as layer thickness and material type, to create walls tailored to specific design requirements and climatic conditions. In addition, FDM supports rapid prototyping, which allows for different insulation solutions to be tested and optimized at the design stage, speeding up the process of bringing innovative building solutions to market [28,29,30].

The resolution and surface smoothness of prints may not match other 3D-printing techniques, such as Stereolithography (SLA) or Selective Laser Sintering (SLS), which may be crucial in applications requiring high-detail accuracy. Moreover, complex designs with large overhangs or delicate structures may require the use of additional supports, which need to be removed after printing, potentially complicating the finishing process. Despite these limitations, FDM remains one of the most accessible and versatile 3D-printing technologies, offering a quick and efficient solution for a wide range of applications from prototyping to small-scale production. Its continuous development, along with the emergence of new materials and technical improvements, promises further expansion of the possibilities and applications of this fascinating additive manufacturing technology [31,32].

### 2.4. Selective Laser Sintering (SLS)

Selective Laser Sintering (SLS) is an advanced 3D-printing technique that enables the creation of durable and complex objects by selectively sintering powdered materials using a laser. This technology allows for the production of strong and durable parts that can be used as structural elements or functional components in construction. SLS is valued for its ability to create complex geometries without the need for additional supports, which is particularly useful in producing custom structural elements such as special connectors or facade elements [33]. This process begins with the preparation of a digital 3D model, which is sliced into thin, horizontal sections by specialized software. 

**Figure 4:**
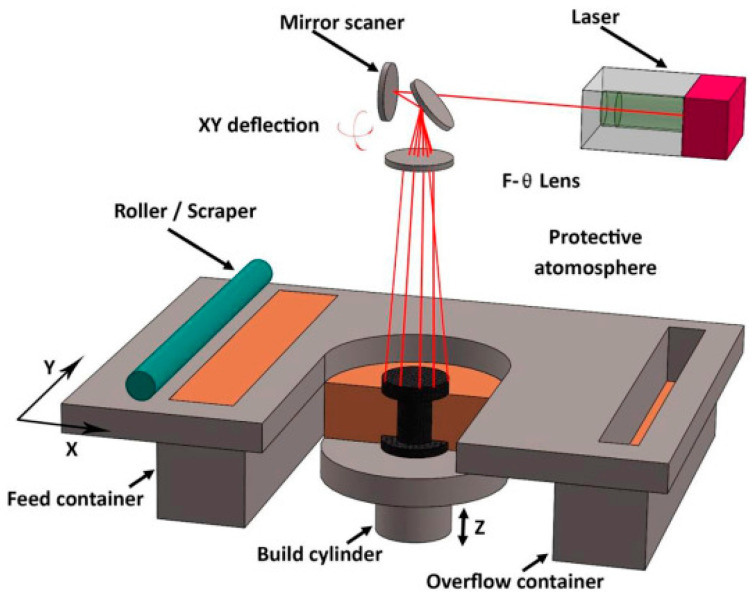
Schematic of SLS 3D-printing process which locally fuses the polymer powder on a preheated platform and accordingly improves the mechanical and microstructural properties of the fabricated parts [35].

Unlike FDM technology which uses filament materials, SLS employs fine powder which can be made from various materials, including polymers such as nylon, polyamides, and metals and their alloys. In an SLS printer, a thin layer of powder is evenly spread on the build platform. Then, a high-power laser is precisely directed onto the powder according to the 3D model data, sintering the material in areas corresponding to the model sections. After sintering the first layer, the build platform is lowered by the thickness of one layer, and a new layer of powder is spread over the surface. The process is repeated until the entire object is built. The use of the SLS method makes it possible to create complex geometries without the need for additional support structures, as the unsintered powder around the object naturally serves as support. This allows for great design freedom and eliminates the need for time-consuming removal of supports after printing, often required in other 3D-printing techniques [34]. SLS stands out for its ability to produce functional parts and prototypes with high strength and durability, making this technology valued in the automotive, aerospace, and consumer goods manufacturing industries. The use of SLS as a 3D printing methodology is presented in Figure 4.

Objects produced by the SLS method are characterized by good mechanical and thermal properties, making this technology particularly useful for engineering and production applications. Despite its many advantages, SLS also has some limitations, including the high costs of equipment and consumables, as well as the need for post-processing cleaning and finishing of printed objects. However, continuous development and innovations in this field contribute to the expansion of SLS capabilities, making this technology increasingly accessible and versatile [22]. 

### 2.5. Stereolithography (SLA)

**Figure 5:**
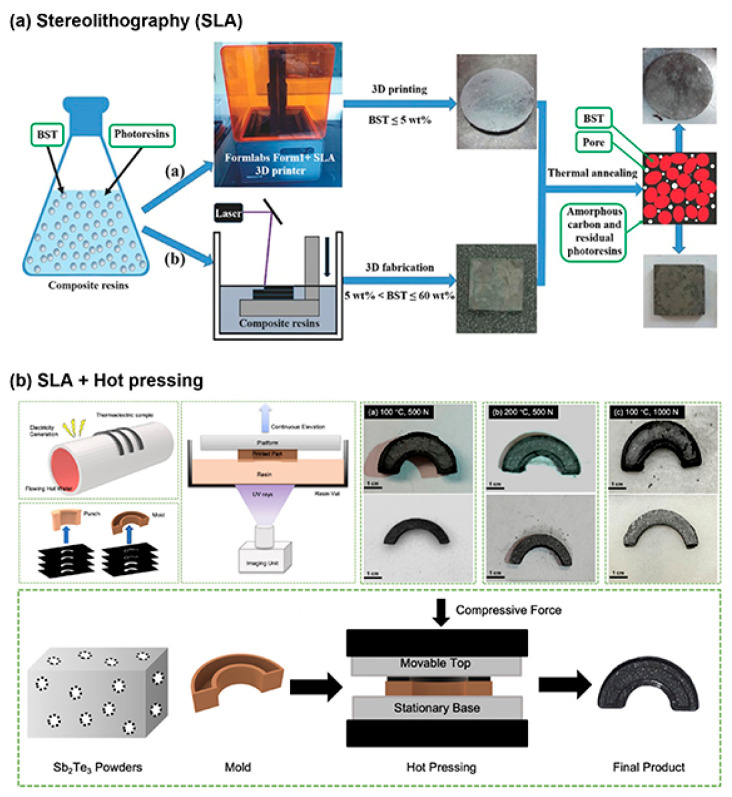
Stereolithography (SLA)-printed TE materials [37].

Stereolithography (SLA), the first developed 3D-printing technology, uses UV light to cure liquid resins layer by layer, creating highly detailed objects (Figure 5). SLA allows for the production of objects with a high degree of detail and surface smoothness. This method, characterized by exceptional accuracy, has found widespread application in many fields, including construction, where its precision is invaluable in creating complex architectural models and structural details. With SLA, it is possible to reproduce complex geometries with unprecedented precision, making this technology indispensable in designing formwork and molds [36]. 

In construction, SLA is used to create molds for concrete or gypsum elements, enabling the realization of projects that require special shapes and details. The ability to create accurate molds and matrices opens up new possibilities for architects and engineers in designing and implementing complex architectural elements that are difficult or impossible to achieve using traditional methods. SLA, with its unique properties, has become a key technology in the field of prototyping and small-scale production, offering solutions that combine aesthetics and functionality. The use of this technology in construction represents a step forward towards a more innovative and precise approach to the design and implementation of construction projects, allowing for the creation of more complex and aesthetically advanced structures [38]. Each of these 3D-printing technologies brings unique capabilities to the construction industry, enabling innovations in design, optimization of production processes, and exploration of new forms and materials. FDM, SLS, and SLA, despite differences in methods and materials, collectively contribute to the evolution of the construction sector, paving the way for more sustainable, efficient, and creative construction practices [39].

### 2.6. Concrete 3D Printing in Construction

Concrete 3D printing in construction, also known as concrete incremental manufacturing, is an innovative technology that transforms traditional construction methods by introducing modern manufacturing techniques. Unlike conventional construction methods, which rely on masonry or concrete rigid forms, concrete 3D printing enables the precise creation of complex structures without the need for traditional molds or formwork. The basis of 3D concrete printing is the use of special printers that apply layers of concrete mix in precisely planned patterns. This process is usually performed by employing one of the main methods such as extrusion, fragmentation, filling, milling, and application. Three-dimensional printing with concrete offers a number of advantages, including the ability to create complex and custom forms that are difficult to achieve with traditional construction methods. The technology saves material, minimizing waste by precisely applying concrete only where it is needed. In addition, the process can significantly reduce construction time through automated production, speeding up construction projects [40,41,42]. However, despite its many advantages, 3D printing with concrete also faces challenges. These include the need to develop suitable concrete mixtures, which must have optimal properties for the printing process such as adequate viscosity, setting time, and strength. Another problem is technological limitations related to the size of printers and the need to adapt designs to the specifics of the machines. In addition, issues of construction standards and regulations are still under development, which can lead to regulatory uncertainty regarding the use of this technology. In practice, 3D concrete printing is used in a variety of construction projects, from residential buildings to infrastructure components. Examples include the construction of residential homes, bridges, and even monuments. Contemporary projects show that the technology has the potential to revolutionize construction by making it more efficient and flexible [29,43,44]. Literature references for each 3D printing technique are presented in Table 2.

## 3. Application of 3D Printing in Manufacturing Highly Insulated Walls

The application of 3D printing to manufacture highly insulated walls represents an innovative approach that changes traditional construction methods and opens up new possibilities in terms of building energy efficiency. The use of this technology allows for the application of advanced insulation materials that can be precisely laid within the wall structure, ensuring optimal thermal insulation [57].

### 3.1. Overview of Insulation Materials Used in 3D Printing

**Figure 6:**
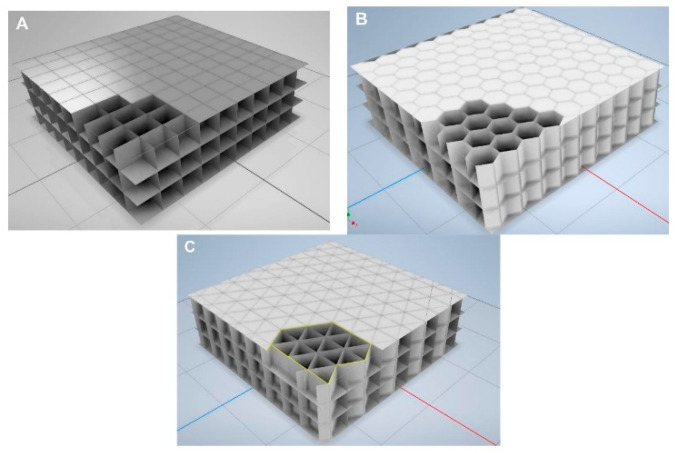
Example of insulation materials made in 3D-printing technology (**A**) square shape, (**B**) hexagonal shape, (**C**) triangular shape [59].

In the context of 3D printing in construction, the use of advanced insulation materials opens up new possibilities for building energy efficiency. Due to the precision offered by 3D printing, it is possible to create walls with integrated insulation channels and spaces for installations, enhancing their functionality and energy efficiency [28,58]. Among the insulation materials used in 3D printing are specialized concrete mixes containing insulation additives, lightweight polymers with aerogel admixtures, and honeycomb structure composites. These materials are characterized by low thermal conductivity and high resistance to external conditions, which is crucial for maintaining optimal thermal insulation of buildings. An example of insulating materials in 3D printing is presented in Figure 6.

Three-dimensional printing also enables the exploration of new forms of insulation materials, such as geopolymers or innovative composites, which may offer better insulation properties while minimizing environmental impact [60,61]. Case studies and pilot projects provide valuable data on the performance, costs, and application possibilities of these materials in practice, showing how 3D-printing technologies can be used to construct homes and buildings with significantly lower energy demands. Three-dimensional printing in construction offers promising prospects for both the environment and economic and technological efficiency, but it also requires overcoming existing technical, regulatory, and market barriers to fully exploit its potential. Future development directions in insulation materials focus on innovations aimed not only at improving thermal efficiency but also at enhancing the functionality of these materials, opening up new possibilities for sustainable and innovative construction [1,62,63]. Examples of specialized concrete mixtures containing insulating additives are presented in Table 3.

### 3.2. Case Studies: Examples of High-Insulation Walls Using 3D Printing

**Figure 7:**
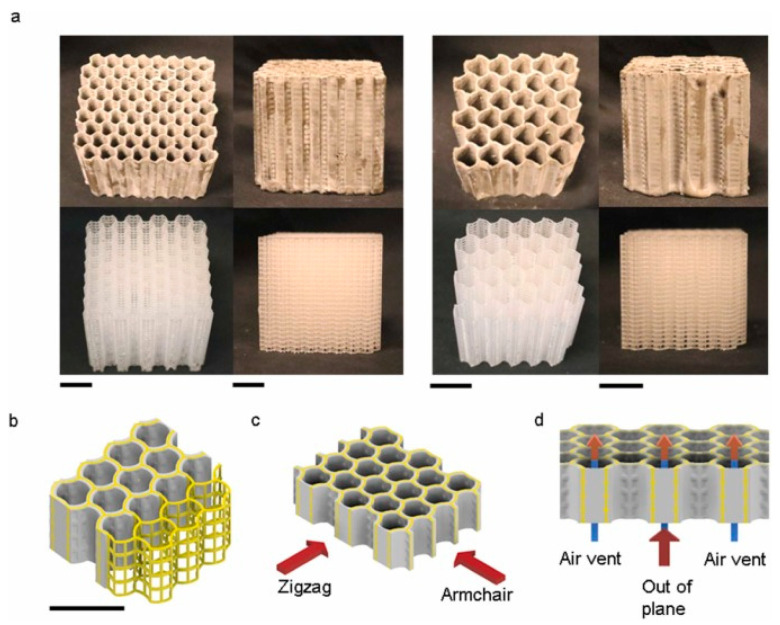
Introduction of HSCC samples (Composite structure, physical properties, and multiphase heat conduction mechanism). (**a**) Images of scaffolds and cast honeycomb samples with relative densities of 0.306 (left four) and 0.165 (right four), including top view and side view. (**b**) Schematic showing the scaffold coated by cement paste (relationship between scaffolds and casted structure). Schematic defining the heating directions. (**c**) Zigzag and Armchair directions. (**d**) Out-of-plane direction [86].

Case studies on the implementation of high-insulation walls using 3D printing present impressive examples where this innovative technology is used to construct homes and buildings with significantly lower energy demands. Three-dimensional printing allows for the application of advanced insulation materials that can be precisely laid within the wall structure, ensuring optimal thermal insulation. The precision of 3D printing provides the ability to create walls with integrated insulation channels and spaces for installations, further increasing their functionality and energy efficiency. Examples of high-insulation walls constructed using 3D printing in construction demonstrate how modern technologies can contribute to improving the energy efficiency of buildings. One such example is the use of gypsum mixtures in the construction of low-rise residential buildings, described by Kuznetsov and co-authors [84]. This technology enables the construction of walls and partitions that provide a high level of living comfort and effective thermal insulation, allowing for the maintenance of a stable interior temperature without excessive energy loss. Another interesting approach is the development of 3D concrete printing with topological information, developed by Lin, Bayramvand, and Meibodi [85]. This method allows for the optimization of the topology of load-bearing walls, which can significantly improve their thermal insulation. With 3D printing, concrete structures can take on complex geometries, which not only increase structural strength but are also optimized for better thermal insulation. An example of honeycomb insulating material is presented in Figure 7.

Fragnito, Iasiello, and Mauro focused on using topology optimization for designing 3D-printed walls with high thermal resistance. Such innovative approaches allow for minimizing air recirculation within walls, translating into better insulation properties [87]. Research on dense and foamed mortars based on cementitious and gypsum-cement binders, conducted by Chernysheva, Shatalova, Lesovik, and others, demonstrates the potential of these materials in the context of 3D printing. The development of such materials could have a significant impact on the thermal insulation of walls while offering high structural strength [88]. Finally, load-bearing systems with a honeycomb structure, described by Qiao, Ren, Wu, and Chen, exemplify how cement composites can be used in walls with high heat resistance. The honeycomb structure not only increases structural strength but also significantly improves the thermal insulation of walls [86]. These examples demonstrate how 3D printing opens new possibilities in designing and implementing high-insulation walls, offering opportunities for material and structural optimization, which is crucial for the sustainable development of the construction sector. Comparative analysis with traditional methods of building insulation walls shows that 3D printing offers significant benefits. It allows for more complex geometries and the integration of various functions into a single construction element, which would be difficult or time-consuming with traditional methods. Moreover, 3D printing minimizes construction waste, providing an additional advantage from a sustainable development perspective.

## 4. Advantages and Challenges

**Figure 8:**
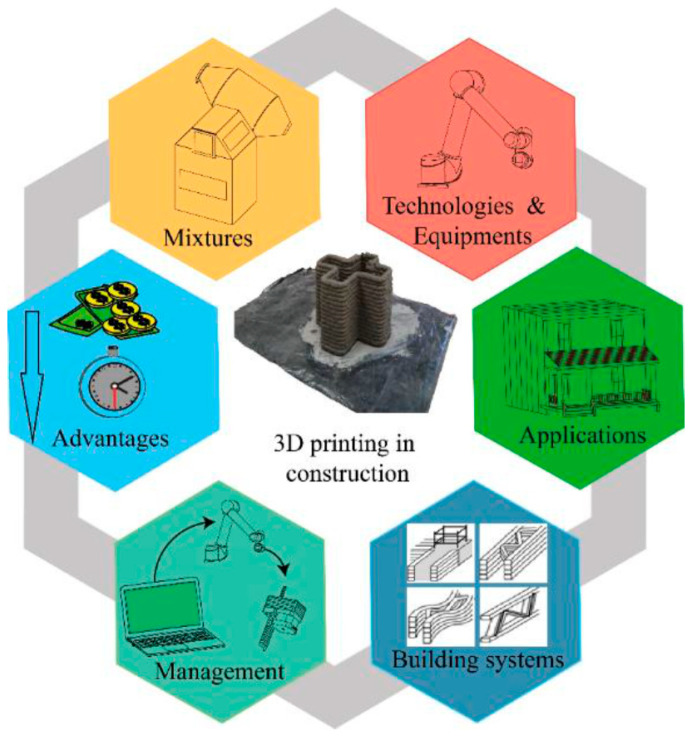
Factors of building construction process using 3D-printing technology [19].

Three-dimensional printing in construction brings significant environmental, economic, and technical benefits that revolutionize traditional building methods and open up new possibilities for the entire industry. The environmental advantages of this technology stem from its ability to minimize waste by precisely using materials, which reduces overconsumption and the production of unnecessary leftovers [89]. Additionally, 3D printing enables the use of sustainable and eco-friendly materials, contributing to the reduction in the construction industry’s carbon footprint. From an economic perspective, 3D printing offers the potential to lower construction costs by reducing the need for manual labor, increasing production speed, and reducing the time and costs associated with material transportation, leading to a more efficient building process. Technical benefits include the ability to create complex geometries and custom designs that are difficult to achieve using traditional methods. Three-dimensional printing also provides high precision and repeatability, crucial for the quality and durability of constructions [90,91]. The possibility of integrating various building functions, such as insulation or installations, in a single production process further enhances the technical efficiency of this method. As a result, 3D printing in construction represents a promising solution that harmonizes environmental needs with economic expectations and technical requirements, pointing the way to the future of sustainable and innovative construction [92,93,94]. While 3D printing in construction offers a range of benefits, it also faces technical, regulatory, and market challenges that must be addressed for this technology to be widely adopted in the industry. Technical challenges often relate to limitations concerning print size and speed, which can impact the scalability and efficiency of large-scale construction projects [95]. There is also a continuous need for the development of printed materials that must meet strict construction standards in terms of strength, durability, and safety. Regulatory challenges arise from the lack of clear regulations and standards for the use of 3D printing technology in construction, leading to legal and technical uncertainty among investors and contractors [96,97,98]. To address these issues, it is essential to advance regulatory frameworks that provide clear guidelines for the implementation of 3D-printing technology. This includes establishing specific standards for materials, processes, and quality control in 3D-printed construction. Engaging with regulatory agencies to develop and refine these standards, as well as promoting industry-wide compliance and certification programs, can help mitigate legal and technical uncertainties. Effective regulatory strategies will facilitate smoother integration of 3D printing into the construction industry, supporting its growth and acceptance. Sections related to 3D printing in construction are presented in Figure 8.

The construction industry, which relies on proven methods and materials, requires regulations that are regularly updated to reflect advances in 3D-printing technology. Finally, market challenges include the need to build awareness and trust among all market participants, from investors to end users. Many people may still be unaware of the possibilities offered by 3D printing or may have concerns about its reliability and performance compared to traditional construction methods. Overcoming these challenges requires joint efforts from researchers, entrepreneurs, and regulatory bodies to promote innovation, educate the market, and develop standards that enable the full potential of 3D printing in the construction industry to be exploited [99,100]. The development of integrated building systems and advanced automation in the process of manufacturing high-insulation walls using 3D-printing technology is one of the most promising directions in modern construction. By combining precise 3D-printing techniques with intelligent control systems, it becomes possible to create complex building structures that integrate various functions and materials in a single production process. The application of automation in this context allows for the optimization of manufacturing processes, reduction in construction time, and minimization of production errors [101,102]. Integrated building systems based on 3D printing enable the design and implementation of walls with high thermal insulation, where insulation is an integral part of the structure, not just an additional layer applied after construction. This technology allows for precise placement of insulation materials within walls, creating complex structures with air chambers, ventilation channels, and spaces for installations, translating into better energy efficiency of the building. Automation in the manufacturing process, supported by advanced algorithms and 3D modeling software, enables the automatic adjustment of print parameters to specific design requirements, ensuring high precision and repeatability of the process. As a result, it becomes possible to create custom building elements that perfectly meet the needs of a particular project while maintaining high standards of thermal insulation [103,104].

The use of innovative methods to produce highly insulated walls using 3D-printing technology poses a number of scientific and engineering challenges that need to be solved. One of the key issues is the development of suitable materials that not only meet thermal requirements but are also compatible with 3D-printing technology. Achieving adequate mechanical strength while maintaining insulating properties requires careful optimization of the compound composition, including the use of innovative additives such as nanomaterials or aerogel-enriched polymers [105,106,107,108]. Another challenge is precise modeling and control of the printing process to ensure consistent quality and structural integrity throughout the structure. In particular, it is crucial to avoid the formation of thermal bridges that could reduce the insulating efficiency of the walls [109,110,111]. The scalability of these technologies and their integration with existing construction methods is also an important issue, requiring comprehensive research into the interactions between printed components and traditional building materials [112,113,114]. In addition, work on automation and robotization of manufacturing processes is needed to increase efficiency and reduce the cost of manufacturing such advanced structures.

The integration of building systems with 3D-printing technology also paves the way for the introduction of intelligent solutions in structures, such as sensors monitoring the technical condition of the building, energy management systems, or active elements that can respond to changes in external conditions, further improving energy efficiency and user comfort. However, further refinement of the technology, development of building materials compatible with 3D printing, and adaptation of building regulations are challenges that remain to be addressed to enable wider use of these innovative solutions in practice. Nevertheless, the potential of integrated building systems and automation in the manufacturing process using 3D printing points to a future where construction becomes more sustainable, efficient, and tailored to individual user needs. Three-dimensional printing in construction offers promising prospects for both the environment and economic and technological efficiency, but it also requires overcoming existing technical, regulatory, and market barriers to fully exploit its potential.

## 5. Future Development Directions

The current market acceptance of 3D-printed structures in the construction sector is still in a nascent stage, with varying responses from stakeholders including investors, contractors, and regulatory oversight bodies. Three-dimensional-printing technology in construction, while offering significant benefits such as cost reduction, reduced construction time, and the ability to realize complex shapes, faces numerous challenges that limit its widespread implementation. Increasing market acceptance of the technology requires comprehensive strategies that include both educational efforts and regulatory changes. Raising awareness of the potential benefits and effectiveness of 3D printing through use case demonstrations and pilot projects that can serve as evidence of the technology’s effectiveness and cost-effectiveness is key. It is also important to develop standards and regulations to ensure compliance and safety of 3D-printed structures, which will help minimize implementation risks and strengthen investor confidence. In addition, it is necessary to support innovation and research into 3D-printed materials, which can increase their attractiveness by improving mechanical and functional properties. An integrated marketing approach that highlights the successes of 3D-printing technology in the construction industry and collaboration with key stakeholders, including regulators and research institutions, can significantly accelerate the adoption and acceptance of this innovative technology in a wide range of construction projects.

Future development directions in insulation materials focus on innovations aimed not only at improving thermal efficiency but also at increasing the sustainability and functionality of these materials. A modern approach to insulation includes the development of materials with variable porosity, which can adjust their thermal properties to changing external conditions, offering optimal insulation depending on the needs [115,116]. These innovations may also include the development of insulation materials with embedded sensors that monitor and regulate the microclimate inside buildings, contributing to increased user comfort and reduced energy consumption [117,118]. One of the promising directions is the development of aerogels—materials with exceptionally low density and high porosity, offering excellent insulation properties while maintaining minimal thickness [119,120]. Aerogels can be applied in the form of panels or sprays, making them exceptionally versatile in construction applications. However, it is important to note the challenges associated with aerogels, such as potential issues with leakage, which could compromise their performance and application in certain environments. Additionally, their inherent fragility and low mechanical strength pose risks of structural damage during transportation, installation, and use, necessitating the development of methods to reinforce or protect aerogels within composites. Another critical issue is the hygroscopic nature of aerogels, where their ability to absorb moisture from the environment can degrade their insulating properties over time, leading to decreased efficiency and potential material failure. Another breakthrough is insulation materials based on nanotechnology, which, as a result of the use of nanoparticles and nanofibers, can offer not only better thermal properties but also greater strength and durability [121,122,123]. Progress in the field of bio-insulation, i.e., insulation materials of plant or animal origin, also indicates future development directions. Materials such as wood wool, cork, sheep’s wool, or mycelium-based insulation are being researched for their ability to provide effective insulation while minimizing environmental impact. Their biodegradability and potential for carbon dioxide sequestration make them attractive from sustainable construction perspective [124,125]. The development of integrated materials that combine insulation with other functions, such as energy generation (e.g., through integrated photovoltaic panels) or self-cleaning, opens up new possibilities for smart facades and walls that not only insulate but also actively contribute to the energy balance of buildings. The future of insulation materials looks promising owing to continuous innovations that not only raise the bar in terms of thermal efficiency but also open new possibilities in terms of functionality and sustainable development in construction [124,126]. Future research in 3D printing of insulating walls should focus on several key areas. New printing materials should be developed that combine excellent insulating properties with high mechanical strength and weather resistance. It is also important to optimize printing parameters, such as speed and accuracy, to improve the efficiency of manufacturing large building components. Research should also include the long-term durability of insulating walls, their resistance to environmental conditions, and their impact on sustainability by evaluating the life cycle of materials and potential recycling. In addition, integrating 3D-printing technology with intelligent microclimate monitoring and management systems can increase energy efficiency and occupant comfort.

## 6. Conclusions

Innovative methods of manufacturing high-insulation walls are crucial for the future of construction, as they contribute to the creation of more energy-efficient and sustainable buildings. The development of these methods, including advanced technologies such as 3D printing, enables the construction of walls with high-performance insulation materials, which can significantly reduce the energy demand for heating and cooling living and commercial spaces. With the precision and flexibility provided by modern technologies, it becomes possible to integrate thermal insulation directly into the wall structure, optimizing the thermal properties of the building as a whole. This integrated approach not only improves energy efficiency but also allows for the creation of structures with complex shapes and advanced functionalities, such as built-in ventilation channels or spaces for installations, which until recently were difficult to achieve with traditional construction methods. Innovations in building materials, including the development of lightweight composites, aerogels, and insulation materials based on nanotechnology, offer new possibilities in designing high-insulation walls. These new materials not only improve thermal insulation but also contribute to increased durability, resistance to atmospheric factors, and the overall environmental impact of buildings. The significance of these innovations extends beyond technical and environmental aspects, also impacting the economic side of construction. By reducing operational costs associated with heating and cooling, investments in high-insulation walls can bring long-term savings for building owners and users and contribute to an increase in property values.

In summary, innovative methods of manufacturing high-insulation walls are an indispensable element of the future of sustainable construction. They not only meet the growing demands for energy efficiency and sustainable development but also open new possibilities for the design and implementation of modern living and commercial spaces that are both comfortable and environmentally friendly.

## Figures and Tables

**Table 1:** Comparison between traditional methods and 3D printing in construction.

Criteria	Traditional Construction Methods	3D Printing in Construction
Cost	Often higher due to materials, labor, and construction time.	Initial costs for equipment and materials may be high, but lower labor and material costs in the long run.
Construction Time	Usually longer, requiring more time for preparation and building.	Reduced construction time due to automation and rapid production.
Precision and Accuracy	Limited by manual work and rigid forms.	High precision due to computer-controlled printing processes.
Customization	Limited, changes are costly and time-consuming.	High, allows for easy modifications to design.
Material Waste	Often significant material waste.	Minimal waste due to the additive nature of the process.
Geometric Complexity	Limited by construction methods and contractor skills.	Capable of creating complex structures and geometries.
Strength and Durability	Based on tested methods, though errors in execution can occur.	High quality, but depends on materials and printing technology.
Sustainability	May lead to significant waste and resource consumption.	Better material efficiency, possibility of using eco-friendly materials.
Regulations and Standards	Well-regulated and standardized.	Still evolving regulations and standards for new technologies.

**Table 2:** Reference example on the use of 3D-printing techniques in the construction industry.

Technology	References
FDM	[45,46,47,48,49]
SLS	[50,51,52,53]
SLA	[54,55,56]

**Table 3:** Examples of specialized concrete mixtures.

Category	Type	Description	Application	References
Concrete mixes with insulating additives	Cellular concrete	Lightweight concrete containing air vapors, which provides good insulation properties.	Exterior walls, roofs, floors	[64,65]
	Perlite concrete	Concrete containing perlite, which increases its insulating properties and reduces weight.	Insulation of roofs, walls and floors	[66,67,68]
	Concrete with the addition of lightweight aggregate	Concrete with lightweight aggregate (such as expanded clay), which improves thermal and acoustic insulation.	Partition walls, elevations, floor slabs	[69,70,71,72]
Polymers doped with aerogel	Aerogel-doped polyurethane	Lightweight, durable polymers with excellent insulating properties thanks to an admixture of aerogel.	Building insulation, refrigeration industry	[73,74,75]
	Aerogel-doped epoxy	Durable epoxy polymers enriched with aerogel for better insulation and weight reduction.	Structural components, protective coatings	[76,77,78,79]
Honeycomb composites	Composite panels, sandwich structures	The honeycomb structure provides high strength with low weight.	Aerospace, construction, automotive structures	[80,81,82,83]

## Data Availability

Not applicable.
